# A comparison of methods to assess the antimicrobial activity of nanoparticle combinations on bacterial cells

**DOI:** 10.1371/journal.pone.0192093

**Published:** 2018-02-01

**Authors:** Claire Bankier, Yuen Cheong, Suntharavathanan Mahalingam, Mohan Edirisinghe, Guogang Ren, Elaine Cloutman-Green, Lena Ciric

**Affiliations:** 1 Department of Civil, Environmental and Geomatic Engineering, University College London, London, United Kingdom; 2 School of Engineering and Technology, University of Hertfordshire, Hatfield, United Kingdom; 3 Department of Mechanical Engineering, University College London, London, United Kingdom; 4 Department of Microbiology, Virology, and Infection Prevention Control, Great Ormond Street Hospital NHS Foundation Trust, London, United Kingdom; VIT University, INDIA

## Abstract

**Background:**

Bacterial cell quantification after exposure to antimicrobial compounds varies widely throughout industry and healthcare. Numerous methods are employed to quantify these antimicrobial effects. With increasing demand for new preventative methods for disease control, we aimed to compare and assess common analytical methods used to determine antimicrobial effects of novel nanoparticle combinations on two different pathogens.

**Methods:**

Plate counts of total viable cells, flow cytometry (LIVE/DEAD BacLight viability assay) and qPCR (viability qPCR) were used to assess the antimicrobial activity of engineered nanoparticle combinations (NPCs) on Gram-positive (*Staphylococcus aureus)* and Gram-negative (*Pseudomonas aeruginosa*) bacteria at different concentrations (0.05, 0.10 and 0.25 w/v%). Results were analysed using linear models to assess the effectiveness of different treatments.

**Results:**

Strong antimicrobial effects of the three NPCs (AMNP0–2) on both pathogens could be quantified using the plate count method and flow cytometry. The plate count method showed a high log reduction (>8-log) for bacteria exposed to high NPC concentrations. We found similar antimicrobial results using the flow cytometry live/dead assay. Viability qPCR analysis of antimicrobial activity could not be quantified due to interference of NPCs with qPCR amplification.

**Conclusion:**

Flow cytometry was determined to be the best method to measure antimicrobial activity of the novel NPCs due to high-throughput, rapid and quantifiable results.

## Introduction

Antimicrobial compounds, solutions and procedures are widely used in the biopharmaceutical and healthcare industry for disinfection and decontamination of pathogens from processes, equipment and devices. Therefore, it is important to use quantitative methods that provide an accurate assessment of how effective these antimicrobial strategies are at decontamination. Within the biologics manufacturing industry, it is common to use viral or bacterial products as raw starting materials when developing a new product or device. However, after development, the manufacturer must be able to demonstrate that their processes can remove any contamination and that the products or devices are safe for human use [1]. The ability to assess decontamination processes is also important in healthcare settings, such as hospitals, to help control and prevent the spread of infectious disease. With the increase in multidrug-resistant infections and ineffective antibiotics, preventative methods for disease control within hospitals are in increasing demand. Currently, hospitals employ a wide variety of disinfection and decontamination procedures, which include filtration devices, use of solvents/detergents and heat treatments [2]

Numerous analytical methods are currently being used within these industries to help quantify the antimicrobial effects of these current disinfection compounds and procedures. However, comparison between different methods and results is often difficult due to non-standardised procedures, the variety of available methods and variation in experimental design [3].

Effectively assessing antimicrobial activity is a hotly debated topic with several methods being used. Currently, one of the most common methods used within hospitals and industry to measure antimicrobial activity is the plate count method for microbial enumeration [4,5]. The plate count method involves serially diluting a culture of bacteria to count colony forming units (CFU) on an agar plate. This method is cheap, easy to use and requires minimal training to perform. However, it is time consuming and labour intensive. Furthermore, issues arise when viable but non-culturable (VBNC) cells are present. VBNC cells have intact membranes and genomic material but their metabolic functions might differ from viable, culturable cells and they can no longer grow on standard media [6]. Many cells might enter this state when under stress, such as exposure to an antimicrobial compound or other sub-optimal abiotic conditions. VBNC can pose risks to public health if there has been an underestimation of total viable, pathogenic bacterial cells.

The use of fluorescent dyes (propidium iodide, SYTO^®^9 and propidium monoazide) are used throughout different techniques as a rapid way to distinguish between populations and determining the viability of cells [7,8], but results can often be variable and cannot be used to determine species of bacteria. One method that uses these fluorescent markers is flow cytometry. A common assay is the LIVE/DEAD BacLight viability assay which uses propidium iodide to stain cells with damaged membranes and SYTO^®^9 which can penetrate damaged and intact membranes [8]. This assay can give a quick, comprehensive and quantifiable overview of the bacterial population. However, flow cytometry requires a large, initial investment and extensive training to competently calibrate the instrumentation, set up complex assays and analyse data but methods can be validated to be in line with ISO standards.

Other methods to determine antimicrobial activity which are now more commonly used, such as molecular methods, do not require the culturing of bacteria. Quantitative polymerase chain reaction (qPCR) is now being used to assess the effectiveness of antimicrobial compounds and procedures due to decreasing cost and the ability to generate rapid results. qPCR can detect and quantify the amount of target genomic material present within a sample thus eliminating the requirement to culture cells. To differentiate between live and dead cells, a viability PCR assay can be used [9]. This involves incubating samples with a DNA-binding dye, such as propidium monoazide (PMA) which binds to free DNA (including dead cells with damaged membranes) which interferes with the PCR amplification by inhibition and therefore live cells are only detected and amplified during PCR [10].

Metallic nanoparticles have been used as antimicrobials for centuries [11]. Researchers are keen to develop new, effective antimicrobial compounds and procedures that reduce cost and the requirement for multiple disinfectants and procedures to prevent contamination. It is now widely known that metallic nanoparticles show strong antimicrobial effects whilst remaining non-toxic to human cells [12–15] with silver being used in several applications such as drug delivery, wound repair and catheters [16]. With nanoparticle integration into medical devices and equipment on the rise, NPCs offer a promising solution to help develop broad-spectrum antimicrobial devices to these multi drug-resistant pathogens in both biopharmaceutical and healthcare industries, eliminating the requirement for other, more laborious methods of decontamination.

For this study a combination of metallic nanoparticles (tungsten carbide (WC), silver (AgNP) and copper (CuNP)) were selected to create nanoparticle combinations (NPCs). We chose these NPs due to the strong evidence for AgNP and CuNPs to produce a strong antimicrobial effect against several species of bacteria [12–15]. There is some limited evidence that WC nanoparticles produce an antimicrobial effect [14]. Gram-positive (*S*. *aureus*) and Gram-negative (*P*. *aeruginosa*) bacterial cells were then exposed to the NPCs at different concentrations (0.05, 0.10 and 0.25 w/v %). *S*. *aureus* and *P*. *aeruginosa* are common hospital pathogens that can cause serious illness in immune compromised patients. Many strains of *S*. *aureus* are known to be resistant to multiple antibiotics and the organism is a known contaminant of surgical equipment and medical devices [17,18]. *P*. *aeruginosa* readily forms biofilms and can be fatal to patients that have suffered from traumatic burns or ventilator associate pneumonia (VAP) [19].

Here we have tested our novel NPCs and assessed their antimicrobial activity by performing a comparison of proven current antimicrobial assays. The effectiveness of these combinations was analysed by three different methods: plate count, flow cytometry (live/dead assay) and qPCR (viability PCR assay). The performance of these approaches was assessed through a series of microbiological and analytical techniques.

## Materials and methods

### Nanoparticle preparation

All nano powders including AMNP0, AMNP1 and AMNP2, WC, Ag and Cu were engineered by Qinetiq Nanomaterials^®^ using patented Tesima^™^ thermal plasma technology (Farnborough, UK) [20]. The chemical contents of formulations AMNP 0, 1 and 2 were previously reported and found to contain different ratios of W, C, Ag and Cu. In particular, particle sizes of AMNP 1 and 2 were found to be in a range of 10–20 nm [21].

### Growth of bacterial strains

Bacteria stock cultures of *P*. *aeruginosa* (NCTC 12903) and *S*. *aureus* (ATTC 6538P) were obtained from -80°C freezer stocks containing 30% glycerol. Each stock solution was streaked onto Tryptic Soya Agar (TSA) using a sterile loop and incubated at 37°C for 24 hours. After incubation, a single colony of each strain was grown in Luria broth (LB) and placed on a shaker (150 rpm) for a further 24 hours at 37°C. A 1:100 dilution of the overnight culture of each pathogen and inoculated into NPC preparations at three concentrations 0.25, 0.10 and 0.05 w/v %, in triplicate with LB broth and incubated at 37°C, shaken (150 rpm) for 24 hours. In each of the experiments we included a positive control with no treatment, a positive control of bacteria exposed to 200μg/ml antibiotic Oxytetracycline, and bacteria-free controls of each treatment and negative controls with no treatment of bacterial cells added.

### Plate count

**Fig 1:**
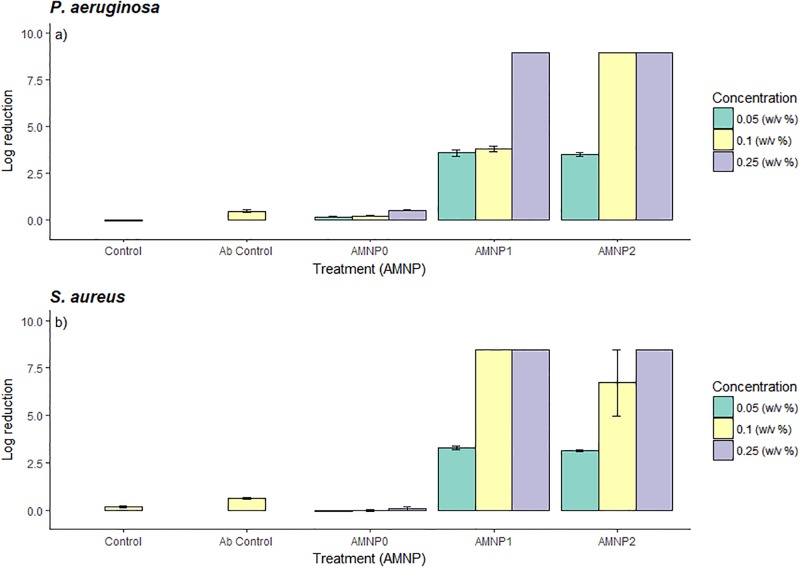
Bar graph showing log reduction for a) *P*. *aeruginosa* and b) *S*. *aureus* for three AMNP nanoparticle composites (AMNP0, 1 and 2) at different NPC concentrations (0.05, 0.10 and 0.25 w/v%) with an antibody control (Ab control). A log reduction of >8-log shows complete inhibition of bacterial growth. Data is expressed as mean (*n* = 3)-/+ SD.

Viable bacterial cell concentrations were estimated by counting CFU’s before and after exposure to the NPCs. This was performed by serial dilution in LB and then removing 10μl of the serially diluted culture and spreading with sterile glass beads (5mm, Sigma, UK) onto an agar plate (Tryptic Soya Agar) in triplicate. The plates were then incubated at 37°C for 24 hours and CFUs were counted. Results for log reduction is shown in Fig 1.

### Flow cytometry

To determine bacterial viability after exposure to nanoparticles by flow cytometry, The LIVE/DEAD BacLight Bacterial viability assay (ThermoFisher, UK) was used. A stock solution of propidium iodide and SYTO^®^9 was prepared according to manufacturer’s instructions. 180μl of the stock staining solution was added to 20μl of diluted sample, in triplicate with appropriate controls, to 1.5ml microcentrifuge tubes and incubated at room temperature in the dark for 15 minutes.

**Fig 2:**
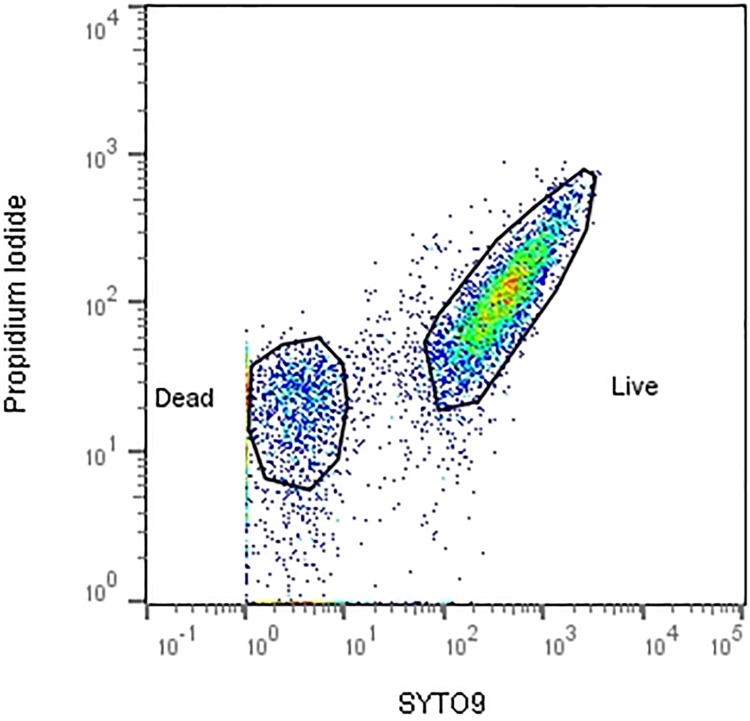
Gating strategy used to determine ‘live’ populations of bacteria after exposure to NPCs (stained positive for SYTO9) and ‘dead’ populations of bacteria (stained positive for propidium iodide). Bacterial populations were gated using positive and negative controls alongside FMO controls. (Gated using FlowJo V10, TreeStar).

Using a calibrated Guava easyCyte^®^ flow cytometer (Merck, UK), the sample was acquired using InCyte software (Merck, UK) and 50,000 events were collected. The bacteria acquisition gate was determined according to forward scatter (FSC) and side scatter (SSC) channels to eliminate background noise and debris. The live and dead populations of bacteria were distinguished by fluorescent channels FL1 (live populations SYTO^®^9) vs FL3 (dead populations PI), as shown in Fig 2. Populations of live and/or dead bacteria were gated according to fluorescence minus one (FMO) controls using single stains of SYTO9 and PI.

### PMA treatment

To determine live and dead populations after exposure to nanoparticle combinations by qPCR, propidium monoazide (Biotium, Inc) was dissolved in 20% DMSO (Sigma, UK) in a light- permeable microcentrifuge tube to a concentration of 20nM. Samples were centrifuged and re-suspended in 500 μl sterile distilled water. 20nM of PMA was added to the re-suspended sample in a transparent microcentrifuge tube and incubated for 5 minutes in the dark. After incubation, samples were placed on ice and exposed to 650W halogen light source for 2 minutes with mixing at a 20cm distance [22].

### DNA extraction

All DNA extractions were performed using QIAamp DNA Mini Kit (Qiagen, UK) extraction kit according to manufacturer’s instructions.

### qPCR

qPCR procedures were performed on Applied Biosystems 7500 Real-time PCR system (Applied Biosystems, UK). All primers and probes for *P*. *aeruginosa* and *S*. *aureus* were based on published literature [23,24] listed in Table 1 and synthesised by Sigma Aldrich, UK. DNA was amplified in 25μl reaction volumes containing 0.1μM of each primer and probe. Amplification and detection was determined using TaqMan Universal PCR Master Mix (x2) (Thermo, UK).

**Table 1:** Primers and probes for sequence amplification and detection (qPCR).

Primer	Target	Sequence	Reference
Probe	*S*. *aureus*	5’ FAM – TAG GCG CAT TAG CAG TTG CAT A – BHQ1 5’	
Primer (F)	*S*. *aureus*	5’ – GTA GAT TGG GCA ATT ACA TTT TGA AGG – 3’	Cloutman-green., (2015)^24^
Primer (R)	*S*. *aureus*	5’ – CGC ATC TGC TTT GTT ATC CCA TGT A – 3’	
Probe	*P*. *aeruginosa*	5′ FAM – AGG TAA ATC CGG GGT TTC AAG GCC – TAMRA 3′	
Primer (F)	*P*. *aeruginosa*	5′ – TCC AAG TTT AAG GTG GTA GGC TG-3′	Schwartz et al., (2006)^23^
Primer (R)	*P*. *aeruginosa*	5′- CTT TTC TTG GAA GCA TGG CAT C-3′	

Briefly, the amplification profile was as follows: 50°C for 2 min, 95°C for 10 min, 40 cycles at 95°C for 15 sec and 40 cycles 60°C for 1 min.

### Statistical analysis

All data are expressed as means ± standard deviation (SD). Statistical analyses were performed using R Studio (v 1.0.136, USA) software, with graphics coded via ggplot2 package. Data was checked for normality (Shapiro-Wilk test). Statistical analyses of data were performed using Students paired t-test or one-way ANOVA with *post hoc* Tukeys and a significant difference is defined as *P <* 0.05.

## Results

To investigate how effective each assay was at determining the antimicrobial effect of the NPCs, three methods were utilised: plate count, flow cytometry (live/dead) and qPCR (viability).

### Plate count

Colonies of *P*. *aeruginosa* and *S*. *aureus* were counted after 24 hours incubation with NPCs at different concentrations (0.05, 0.10 and 0.25 w/v %). Complete inhibition of bacteria at 0.25 w/v% concentration was observed for both *P*. *aeruginosa* and *S*. *aureus* for AMNP1 and 2 (>8-log reduction). At 0.10 w/v % there was also complete inhibition of growth when exposed to AMNP2 for *P*. *aeruginosa* and AMNP1 for *S*. *aureus*. 0.05 w/v% had a reduction of growth but complete inhibition was not observed for either *P*. *aeruginosa* or *S*. *aureus* (~3-log reduction). AMNP0 was not shown to have an effect on growth of either pathogen at all concentrations (<0.5-log) with similar results to the control (no treatment, <0.2 log reduction). Fig 1 shows log reduction values after NPC treatment.

A one-way ANOVA shows a significant difference overall between NPC treatments (AMNP0–2) for *P*. *aeruginosa* and *S*. *aureus*, respectively (Fig 1a: F_*3*,*26*_ = 20.84, *P* = 0.001 and b: F_*3*,*26*_ = 21.75, *P* = 0.001). *Post hoc* Tukeys HSD test showed a significant difference between AMNP0 and all other treatments (*P <* 0.001) with the exception of the control (*P* = 0.99), which was not significant when compared with AMNP0 or AMNP1 with AMNP2 (*P* = 0.99) for both *P*. *aeruginosa* and *S*. *aureus*.

### Flow cytometry

**Fig 3:**
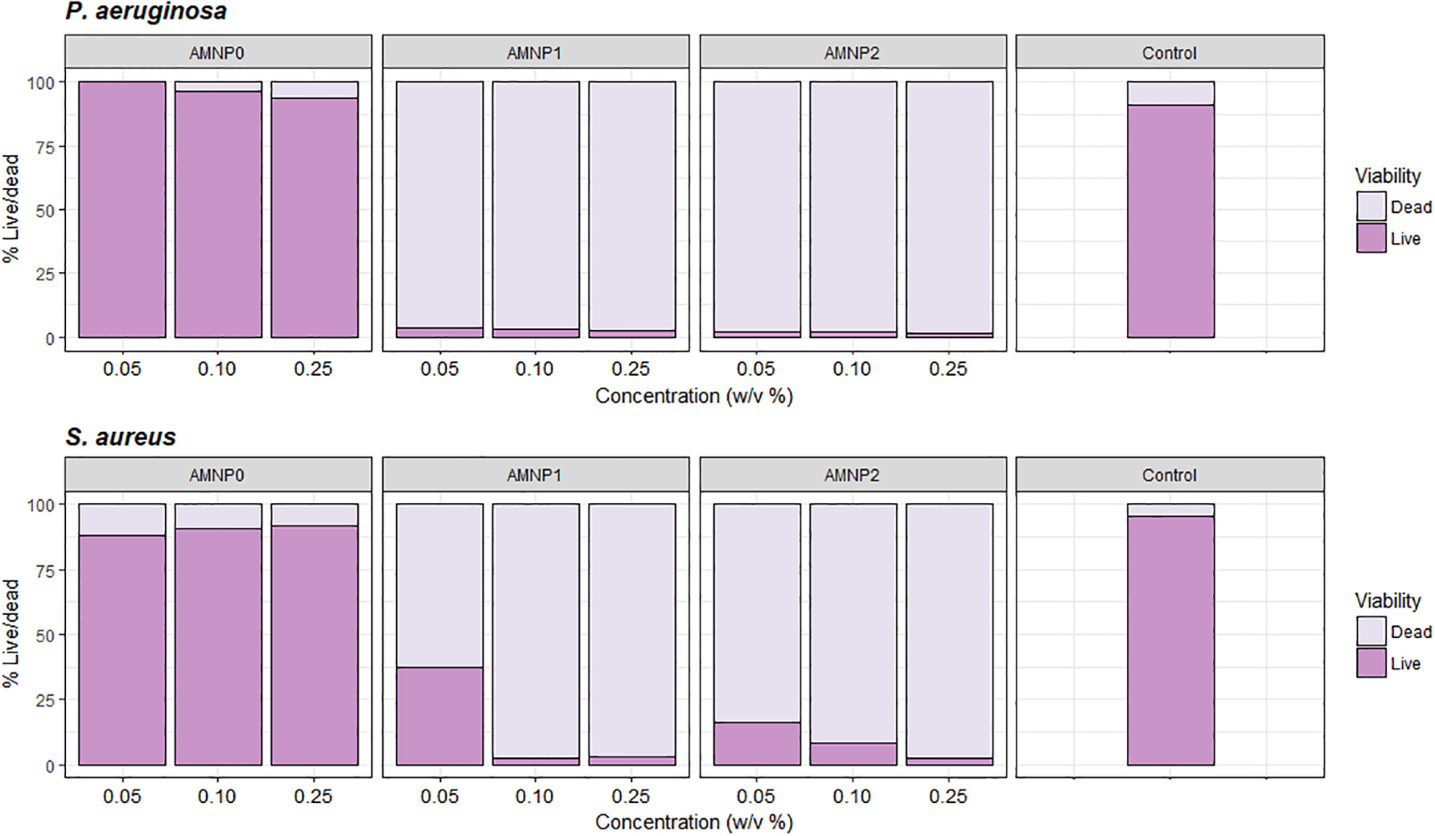
Mean (*n* = 3) flow cytometry data for proportion of live and dead bacterial populations after exposure to NPCs (AMNP0, 1, 2 and antibiotic control (oxytetracycline)) with a) *P*. *aeruginosa* and b) *S*. *aureus*. Proportions of live and dead were calculated using the total absolute cell count by the Guava easyCyte flow cytometer which was determined through gating of the live and dead cell populations (FlowJo V10, TreeStar).

Populations of bacteria acquired by flow cytometry were analysed using FlowJo V10 (TreeStar, USA), gating strategy is shown in Fig 2. Proportions of live and dead bacteria within one population exposed to the NPC treatments were used to indicate the overall viability of the bacterial population before and after exposure (Fig 3). Dark purple bars show ‘live’ populations of bacteria, whilst light purple bars indicate ‘dead’ populations.

A one-way ANOVA was used to analyse differences between treatments and concentrations. A significant difference was shown between all treatments for proportion of live (F_2,6_ = 2284, *P* = 0.001) and dead bacteria (F_2,6_ = 41.58_,_ *P* = 0.001) for *P*. *aeruginosa* and *S*. *aureus*, respectively. Significant differences were shown between AMNP0 and all other treatments (AMNP1 and 2), (*P* < 0.001 for live and dead bacteria for *P*. *aeruginosa* and *S*. *aureus*). No significant difference was shown between treatments AMNP1 and 2 for both live and dead bacteria (*P* > 0.7). Overall, for all concentrations there is an increasing trend of ‘dead’ bacteria as NPC concentration increased for AMNP1 and 2.

### qPCR

To distinguish live and dead populations using qPCR, viability qPCR assay was performed with the addition of propidium monoazide (PMA). PMA blocks amplification of free DNA, i.e. dead cells. ΔCT was calculated by subtracting the mean post-treatment CT values from the mean control CT values. ΔCT values above 0 show an increase in amplification (i.e. more cells with more DNA). ΔCT values below 0 show a decrease in amplification (i.e. fewer cells and less DNA).

**Fig 4:**
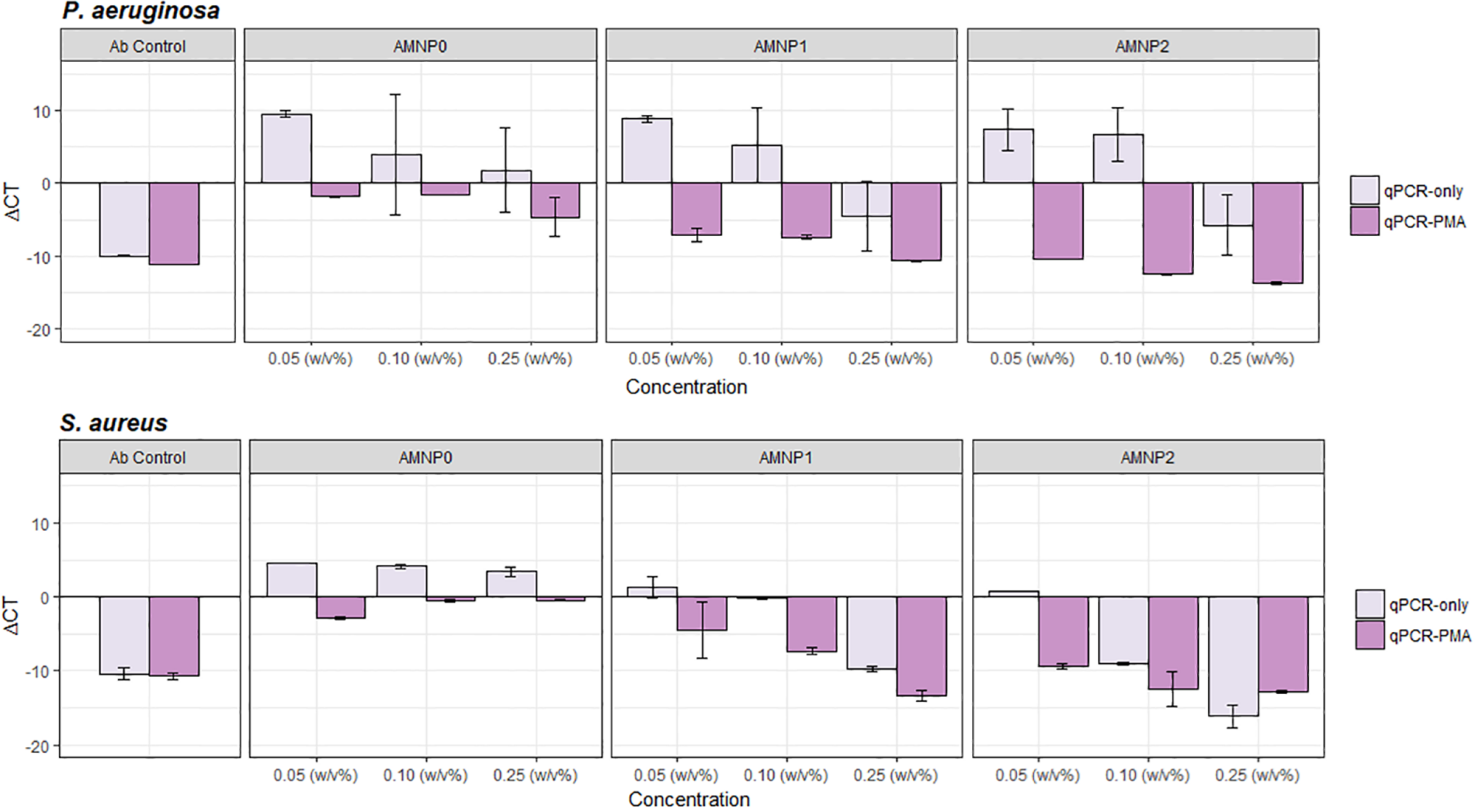
Comparison to determine the effectiveness of qPCR-PMA and qPCR only to distinguish between live and dead cells. *P*. *aeruginosa* (a) and *S*. *aureus* (b) were exposed to different concentrations of NPCs (0.05, 0.10 and 0.25 w/v %) for 24 hours and an Ab control. qPCR-only and qPCR-PMA was used to detect cell lysis via measurement of ΔCT. ΔCT values below 0 show a decrease in amplification (less DNA, dead cells) and ΔCT values above 0 show an increase in amplification (more DNA, live cells). Data expressed as mean (*n* = 3) -/+ SD.

Fig 4 shows a trend for ΔCT values above 0 in the absence of PMA (qPCR-only) when compared with samples treated with PMA for both *S*. *aureus* and *P*. *aeruginosa* (qPCR-PMA).

Typically, positive ΔCT values were also shown for *P*. *aeruginosa* and *S*. *aureus* at lowest concentration of NPC exposure (0.05 w/v%). Negative ΔCT values were observed when pathogens were exposed to higher concentrations of NPCs (0.10 and 0.25 w/v%) suggesting a higher rate of dead cells within the population.

To determine the effect of PMA treatment, a paired t-test was performed. Results show a significant difference for *S*. *aureus* (t_26_ = 6.59, *P =* 0.001) and *P*. *aeruginosa* (t_26_ = 9.49, *P =* 0.001) when comparing qPCR-PMA and qPCR-only.

Furthermore, a one way ANOVA showed a significant difference between NPC treatments (AMNP0–2) for *P*. *aeruginosa* qPCR-PMA (F_*2*,*24*_ = 61.98, *P =* 0.001) and no significant difference with qPCR-only (F_*2*,*24*_ = 0.144, *P =* 0.86). There were also significant differences between NPCs (AMNP0–2) for *S*. *aureus* for both qPCR-PMA (F_2,24_ = 30.01, *P =* 0.001) and qPCR-only (F_2,24_ = 11.2, *P =* 0.001).

*P*. *aeruginosa* shows a prominent distinction of ΔCT values between qPCR-PMA and qPCR-only. Positive ΔCT values occur when *P*. *aeruginosa* with qPCR-only and negative ΔCT values are observed when *P*. *aeruginosa* is exposed to PMA (qPCR-PMA). However, this is not as clear with Gram-positive, *S*. *aureus* which also show negative ΔCT values with (qPCR-PMA) and without (qPCR-only) the addition of PMA.

## Discussion

Methods used for quantifying antimicrobial activity vary in industry and in healthcare. Currently, plate counts, flow cytometry and qPCR are all common methods for assessing antimicrobial activity, however, there is little consistency between assays in industry and the healthcare profession. The plate count method on solid media has been used for several decades [25] and is still common practice. However, with the decreasing cost of more quantitative assays, namely molecular methods, plate count has fallen out of favour as being time consuming and laborious.

Here, various methods of determining the antimicrobial activity of different NPCs at various concentrations (0.05, 0.10 and 0.25 w/v %) were tested. The data indicate that overall, a strong comparable antimicrobial effect could be determined using the plate count method (Fig 1), flow cytometry (Fig 3) and qPCR-PMA method (Fig 4).

The plate count method gave a semi-quantitative assessment of antimicrobial activity of the NPCs by giving an estimate of the overall concentration of live bacteria cells before and after exposure. However, by using flow cytometry, we were able to quantify the proportions of live and dead cells within a bacterial population using LIVE/DEAD BacLight Viability kit (ThermoFisher, UK), which enabled a much more quantitative measurement of cell viability before and after exposure to NPC treatments when compared to plate count estimates. Although flow cytometry is more accurate and quantitative, we see a similar trend in reduction of live bacteria with increasing concentrations of NPCs for both pathogens (with the exception of AMNP0) when comparing flow cytometry with the plate count method.

From qPCR-PMA, results show a similar trend to flow cytometry and plate count as negative ΔCT values (i.e. dead cells) occur when *S*. *aureus* and *P*. *aeruginosa* have been exposed to higher concentrations of NPCs for both AMNP1 and AMNP2. However, plate count and flow cytometry show a clear distinction between AMNP1 and 2 with AMNP0, which shows no antimicrobial activity. With qPCR-PMA, AMNP0 results suggest there might be some antimicrobial activity occurring at higher concentrations, in particular *P*. *aeruginosa* with negative ΔCT values. It is thought that nanoparticles might interfere with the qPCR amplification as they are also known to bind to DNA [26]. PMA also works by binding to free DNA within the sample, including cells that have a damaged membrane [22]. This binding of both the NPCs and PMA might have led to results that show inconsistent data and interference with qPCR amplification. This was also demonstrated in a study by Wang *et al*., (2005) where silicon NPs were proven to interfere with the qPCR amplification [27].

A distinction between positive and negative ΔCT values was observed for *P*. *aeruginosa* with qPCR-PMA treated cells (Fig 4), however, this was not as prominent for *S*. *aureus*. It is thought that this could be due to differences in cell wall structure and ability of the bacteria to uptake charged particles. As *P*. *aeruginosa* cells have wide, non-specific porin channels, this could allow the PMA to penetrate the Gram-negative cells more readily than Gram-positive bacterial cells [28]. Moreover, Gram-negative bacteria like *P*. *aeruginosa*, lack teichoic acids and the peptidoglycan cell wall is much thinner in comparison to Gram-positive bacteria such as *S*. *aureus*. *S*. *aureus* has a much thicker cell wall, made up of several layers of peptidoglycan (up to 100nm thick) with teichoic acids embedded within these layers [29]. This tough exterior of the Gram-positive *S*. *aureus* might have blocked PMA from penetrating the cells as effectively and therefore no clear distinction could be made between qPCR-PMA and qPCR-only treatments.

Combinations AMNP1–2 showed strong antimicrobial activity for both *S*. *aureus* and *P*. *aeruginosa*. Consistently higher inhibition of growth of the Gram-negative organism was shown in comparison to *S*. *aureus* for both plate count and flow cytometry. This could be due to the composition of the bacterial cell wall and ability to resist antimicrobial compounds. It is the increased cell wall strength that is thought to give Gram-positive bacteria a higher level of protection against antimicrobial agents such as certain antibiotics [30].

AMNP0 was constituted of 100% tungsten carbide NPs and demonstrated no antimicrobial activity for plate count method or flow cytometry. qPCR-PMA showed mostly positive (or close to 0) ΔCT values when compared to other combinations, indicating the majority of cells to be live, with (qPCR-PMA) and without (qPCR-only) PMA treatment. A study by Syed *et al*., (2010)[14] showed bacteriostatic activity for tungsten nanoparticles against *S*. *aureus*, thought to be attributed to generation of reactive oxygen species (ROS), however, here, with tungsten carbide, we saw no evidence for antimicrobial activity. This could be due to the size of the nanoparticles that were used in this study, which were larger (10-20nm), compared to Syed *et al*., who used NPs <10nm. Having smaller nanoparticles increases the surface area in which they can disrupt the cell membrane of the pathogen leading to cell destruction [31].

Overall, it is thought that toxicity of the nanoparticles to microorganisms occurs when the nanoparticles disrupt the cell membrane causing free radical formation and reactive oxygen species (ROS) to develop [15], however, this differs between bacterial species and nanoparticles. Some studies suggest silver and copper nanoparticles affect the primary cell wall structure of Gram-positive bacteria (e.g. *Staphylococcus aureus*), in particular the glycan strands and peptide branches [32,33]. However, the mechanisms of this remain unclear. There is limited evidence that tungsten nanoparticles might have some antibacterial effect with one study showing an inhibition of *S*. *aureus* and *E*. *coli* growth after exposure to these nanoparticles [14].

Silver and copper nanoparticles are thought to effect Gram-negative bacteria (such as *P*. *aeruginosa*) by interrupting transcription factors, thus preventing quorum sensing signals that cause biofilm formation and pathogenicity[34,35]. Silver is also known to have potent effects on organisms such as *E*. *coli*, *Pseudomonas spp*. and the protozoan pathogen Cryptosporidium [12,36,37]. When comparing *S*. *aureus*, flow cytometry and plate count methods, the plate count method showed higher rate antimicrobial activity with high log reductions for AMNP1 and 2. However, the same exposure showed lower antimicrobial activity for the same nanoparticle compositions (AMNP1 and 2) when analysed by flow cytometry with a higher proportion of live cells than suggested by the plate count method. As flow cytometry is not affected by viable but non culturable cells, we suggest this could be a reason as to why a slightly higher antimicrobial activity with *S*. *aureus* exposed to AMNP1 and 2 was seen using the plate count method. In contrast to this, the qPCR method for *S*. *aureus* with (qPCR-PMA) and without PMA (qPCR-only) showed negative ΔCT values in comparison when exposed to high concentrations of NPCs which was also shown for AMNP0 qPCR-PMA and qPCR-only, suggesting more live cells were amplified. However, unlike the plate count method and flow cytometry, with qPCR-PMA no distinction could be made as to which NP composite (AMNP0–2) was the best antimicrobial using qPCR only.

Interference from NPCs was not shown with either the plate count method or flow cytometry. Flow cytometry did not show NPC interference throughout the assay when comparing bacteria-free controls containing no NPCs and bacteria-free NPC controls. NPCs remained undetectable in the fluorescence channels.

In summary, this comparison of three commonly used methods to determine antimicrobial activity showed best results were achieved using flow cytometry. This high-throughput method showed no interference by NPCs and allowed distinction between live and dead populations of cells. The plate count method also remained unaffected by the NPCs, however, this method was time consuming due to large volume of samples and less accurate due to the inability to detect viable but non-culturable cells. Similar results were found in a study by Pan *et al*., (2014) who determined flow cytometry to be most accurate when comparing with plate count methods and spectrophotometry [38]. qPCR data was not as quantifiable as plate count or flow cytometry with no clear distinction between live and dead cells when comparing qPCR-only and qPCR-PMA between NPCs at all concentrations. Therefore, it was determined that this was the least accurate method for determining antimicrobial activity of nanoparticles.

The data presented here gives an overall assessment of common methods used in industry and in the healthcare profession for determining antibacterial activity of procedures and processes with use of nanoparticles which could be useful in the food, pharmaceutical and cosmetic industries.

## Supporting information

S1 Table CFU data for log reduction calculations relating to Fig 1. (CSV)

S2 Table Flow cytometry data relating to Fig 3. (CSV)

S3 Table qPCR data relating to Fig 4. (CSV)
